# The J-GRID as a new player of networking

**Published:** 2011-01-10

**Authors:** Yoshiyuki Nagai, Yoshiko Okamoto

**Affiliations:** 1 RIKEN Center of Research Network for Infectious Diseases (CRNID), Tokyo 101-0051, Japan; 2 Japan Science and Technology Agency (JST), Saitama 332-0012, Japan

## 

Infectious diseases heed no national borders. In order to enhance international research collaboration, the government of Japan launched a program in 2005, which is now named the Japan Initiative for Global Research Network on Infectious Diseases (J-GRID). This program includes establishing collaborative research centers in Asian and African countries on a reciprocal basis between Japanese universities/institutions and overseas partner universities/institutions and setting up its headquarters CRNID at RIKEN to connect those bilateral collaboration centers into a network, the J-GRID. The J-GRID now consists of 12 collaboration centers in 8 countries (6 in Asia and 2 in Africa). Here, we describe the mission of J-GRID and some of its research results just coming out. However, the J-GRID is still in its infancy and needs a great deal of effort to maximize its research capacity and to ensure its sustainability. J-GRID is eager to learn a lot from Institut Pasteur Network and other international networks that are proud of long history, high quality research and great contribution to the global public health. What a single network is able to cover is limited. In view of the geographical complementarities, particularly in Asia, close collaboration between different networks (networking of networks) will certainly give rise to a profound synergistic effect.

